# Within-population sperm competition intensity does not predict asymmetry in conpopulation sperm precedence

**DOI:** 10.1098/rstb.2020.0071

**Published:** 2020-10-19

**Authors:** Martin D. Garlovsky, Leeban H. Yusuf, Michael G. Ritchie, Rhonda R. Snook

**Affiliations:** 1 Department of Animal and Plant Sciences, University of Sheffield, Sheffield S10 2TN, UK; 2 Centre for Biological Diversity, University of St Andrews, St Andrews KY16 9TH, UK; 3 Department of Zoology, Stockholm University, Stockholm 106-91, Sweden

**Keywords:** sperm competition, postcopulatory sexual selection, conspecific sperm precedence, demographic history, postmating prezygotic reproductive isolation, speciation

## Abstract

Postcopulatory sexual selection can generate evolutionary arms races between the sexes resulting in the rapid coevolution of reproductive phenotypes. As traits affecting fertilization success diverge between populations, postmating prezygotic (PMPZ) barriers to gene flow may evolve. Conspecific sperm precedence is a form of PMPZ isolation thought to evolve early during speciation yet has mostly been studied between species. Here*,* we show conpopulation sperm precedence (CpSP) between *Drosophila montana* populations. Using Pool-seq genomic data we estimate divergence times and ask whether PMPZ isolation evolved in the face of gene flow. We find models incorporating gene flow fit the data best indicating populations experienced considerable gene flow during divergence. We find CpSP is asymmetric and mirrors asymmetry in non-competitive PMPZ isolation, suggesting these phenomena have a shared mechanism. However, we show asymmetry is unrelated to the strength of postcopulatory sexual selection acting within populations. We tested whether overlapping foreign and coevolved ejaculates within the female reproductive tract altered fertilization success but found no effect. Our results show that neither time since divergence nor sperm competitiveness predicts the strength of PMPZ isolation. We suggest that instead cryptic female choice or mutation-order divergence may drive divergence of postcopulatory phenotypes resulting in PMPZ isolation.

This article is part of the theme issue ‘Fifty years of sperm competition’.

## 1. Introduction

Widespread polyandry in animals presents the opportunity for postcopulatory sexual selection (sperm competition and cryptic female choice) and sexual antagonism to accelerate the coevolution of male ejaculate × female reproductive tract interactions [1–5]. Accordingly, reproductive traits such as gamete cell surface proteins, male seminal fluid proteins, female reproductive tract and sperm morphologies, show elevated rates of molecular and morphological evolution [6–9]. Barriers to gene flow between populations caused by reproductive interactions during or after mating but before fertilization (i.e. postmating prezygotic, PMPZ) are expected to emerge early during speciation due to the rapid codiversification of reproductive traits within populations [10].

Identifying the barriers to gene flow that emerge earliest during reproductive isolation is key to understanding the origin of species [11,12]. Conspecific sperm precedence (CSP) is a widely observed form of competitive PMPZ isolation found where paternity is biased towards conspecifics when a female mates with both a con- and hetero-specific male [13]. CSP can result from postcopulatory selection either via conspecific sperm being more successful in sperm competition and/or favoured by cryptic female choice [5,14,15]. If postcopulatory sexual selection can facilitate the evolution of PMPZ isolation, then asymmetries in the strength of PMPZ isolation acting between taxa may reflect differences in the strength of sexual selection acting within populations. For instance, *in vitro* experiments in mouse (*Mus *spp.**) have shown that CSP between taxa is correlated with the intensity of sperm competition acting within taxa [16,17]. This pattern may not hold if selection against hybridization favouring divergence in prezygotic traits (i.e. reinforcement) and selection on sperm competition are not acting in concert [18], if divergence results from the stochastic fixation of different alleles in different populations, i.e. ‘mutation-order’ divergence [19], or cryptic female choice favours different traits in different populations. Additionally, where females remate, overlapping foreign and coevolved ejaculates within the female reproductive tract might alter PMPZ outcomes. For instance, a foreign male ejaculate could negatively affect the fertilization function of a coevolved male ejaculate, or a coevolved ejaculate could provide the proper postmating female response improving the fertility of a foreign male ejaculate [20].

CSP is thought to evolve early during speciation [21]. In sympatry, CSP can evolve as a reinforcing mechanism [18,22]. The factors shaping the evolution of CSP during, as opposed to after, divergence remain poorly understood. Between divergent allopatric populations, conpopulation sperm precedence (CpSP) has been shown in some species [23,24], while other studies show little or no evidence of CpSP [17,25–27]. Reproductive isolation can evolve in allopatry such that barriers will arise, but the time scale of this, and the role of sexual or other selection versus mutation accumulation is poorly understood [12]. The appearance of isolating mechanisms should reduce gene flow during or following divergence, but the demographic histories of taxa in studies of CpSP are often poorly resolved, hampering inference about the time scale and role of CpSP in nascent reproductive isolation. For instance, it is not currently known whether CpSP can evolve during divergence with extensive gene flow or such incompatibilities only appear or spread after gene flow has ceased. If CpSP does play a role in speciation with gene flow, does it successfully stop gene flow following its appearance?

We have previously described prezygotic reproductive isolation between three populations of *Drosophila montana*, from Crested Butte, Colorado, USA (referred to as Colorado), Oulanka, Finland and Vancouver, Canada (electronic supplementary material, figure S1) [28,29]. All three populations show premating and PMPZ isolation (electronic supplementary material, figure S1) but no postzygotic isolation [28] so reproductive traits are unlikely to have diverged due to reinforcement. If reproductive isolation is solely due to isolation by distance, then the more distant Finnish population should be most divergent from the two North American populations and exhibit stronger reproductive isolation. However, total reproductive isolation is strongest between the geographically closer Colorado and Vancouver populations suggesting reproductive isolation has evolved more quickly between these populations [28]. Vancouver females discriminate against Colorado males and both populations show non-competitive PMPZ isolation, where females successfully store sperm from foreign males after mating but lay many unfertilized eggs. PMPZ isolation is strongest when Colorado females mate with Vancouver males [28,29].

Using the *D. montana* system, we set out to ask (i) what is the relative time scale of divergence and is there evidence for gene flow during divergence? And more recently, (ii) do populations show CpSP and if so, is it concordant with non-competitive PMPZ isolation? (iii) Is the strength of PMPZ isolation predicted by the strength of postcopulatory sexual selection acting within populations? And (iv), do overlapping foreign and coevolved ejaculates interact to alter PMPZ outcomes?

## 2. Methods

### (a) Fly stocks

Mated female *D. montana* were collected from riparian habitats in Crested Butte, Colorado, USA (38°49′ N, 107°04′ W; referred to as Colorado), Oulanka, Finland (66°22′ N, 29°20′ E) and Vancouver, Canada (48°55′ N, 123°48′ W). Population cages were established by combining 20 F3 males and females from each of 20 isofemale lines (800 flies total per population) in 2008 (Oulanka and Vancouver) and 2013 (Colorado population used for measuring reproductive isolation and reproductive investment) [28,29]. The Colorado population cage used for Illumina sequencing was established in 2009 from 13 isofemale lines (520 flies total) [28]. All stocks were maintained in large outbred populations on Lakovaara malt media [30] in overlapping generations in constant light at 19°C to prevent females entering reproductive diapause. Flies were collected within 3 days of eclosion and kept in single-sex vials of 10–20 individuals until reproductive maturity at 21–28 days old.

### (b) Demographic modelling

#### (i) Sequencing, alignment and allele frequency estimation

Previous estimates of population divergence between these populations were based on a few mtDNA and microsatellite markers [31]. To gain more accurate estimates of divergence time and the extent and time scale of ongoing or historical gene flow, we performed explicit demographic modelling of the focal populations using whole-genome Pool sequencing data [32] and calculated within population genetic diversity. We obtained trimmed Pool-seq data for the three populations from Parker *et al.* [33]. Genomic DNA was extracted from pooled samples of 50 females per population and sequenced using an Illumina HiSeq 2000 (see electronic supplementary material). We generated three-dimensional joint-site allele frequency spectra (3D-AFS) and compared the fit of the spectra against competing demographic models using ∂a∂I with an existing pipeline [32,34,35]. We tested seven demographic models for the history of these populations (table 1; electronic supplementary material, figure S2), which we fitted to the 3D-AFS and performed three rounds of model optimizations using the Nelder–Mead method and estimated log-likelihoods using a multinomial approach (see electronic supplementary material). We performed model evaluation by calculating Akaike information criterion (AIC) for each model run [32].

**Table 1.** Results from demographic modelling using ∂a∂I comparing the three-dimensional-allele frequency spectra on a dataset of 8000 SNPs (see also figure 1). For detailed description of models tested, see electronic supplementary material. log-lik, log likelihood; AIC, Akaike Information Criterion; *θ*, nucleotide diversity; nuOul, nuVan, nuCol, effective population size in Oulanka, Vancouver and Colorado, respectively; nuAm effective population size of precursor North American population; mA, ancient migration between Oulanka and precursor North American population; m1, ancient migration between Vancouver and Oulanka; m2, ancient migration between Vancouver and Colorado; m3, ancient migration between Oulanka and Colorado; initial and secondary divergence are represented by T1(a) and T2, where T1(b) and T3 correspond to the start or end of periods of isolation. Population splits are denoted by T1 (between Oulanka and North America) and T2 (between Vancouver and Colorado). T1(a) and T1(b) denote the start or end of periods of migration following the split between Oulanka and North American populations. T3 denotes start or end of periods of migration following the split between Vancouver and Colorado.

model	log-lik	AIC	*θ*	nuOul	nuAm	nuVan	nuCol	mA	m1	m2	m3	T1(a)	T1(b)	T2	T3
(1) No-migration	−24429.7	48871.4	194.07	0.170	4.439	5.392	4.522					3.525		3.106	
(2) Full symmetrical migration	−8923.31	17866.62	1397.29	0.243	0.473	0.237	0.551	0.317	0.785	4.486	0.603	0.875		11.810	
(3) Adjacent migration	−8668.5	17355	201.46	2.366	0.686	1.889	2.435	4.542	0.126	0.771		0.490		25.528	
(4) Period of ancient migration after initial divergence between North American populations and Oulanka	−9444.58	18903.16	1326.13	0.342	0.550	0.601	0.657	0.629				1.753		0.086	
(5) Ancient migration persisting after divergence between Colorado and Vancouver populations	−9931.74	19879.48	538.23	1.249	1.017	2.080	2.058	1.535				9.290	0.163	0.224	
(6) Short period of secondary contact beginning after divergence and a period of isolation between Colorado and Vancouver	−9285.95	18589.9	420.16	1.269	0.372	1.372	0.948		0.235	2.109		3.487		0.487	3.186
(7) Secondary contact beginning during divergence between Colorado and Vancouver	−8747.33	17510.66	363.67	0.742	0.182	0.913	1.396		0.336	1.307		26.891		9.654

**Figure 1.**
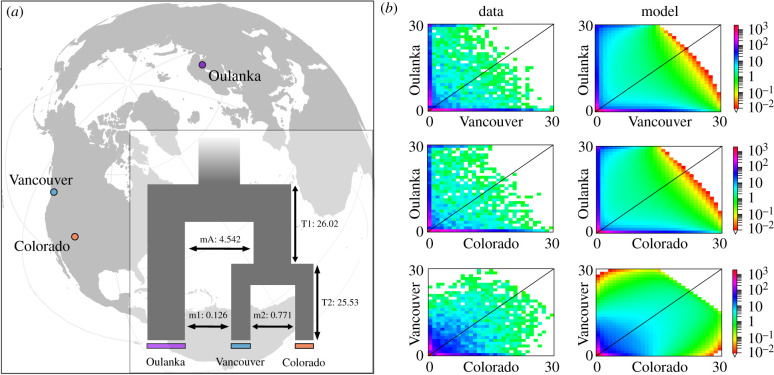
(*a*) locations of *D. montana* populations. Inset: graphical representation of best fit model; adjacent migration between populations (table 1). mA, ancient migration between Oulanka and precursor North American population; m1, ancient migration between Vancouver and Oulanka; m2, ancient migration between Vancouver and Colorado; T1, divergence between Oulanka and precursor North American population; T2, divergence between Colorado and Vancouver. (*b*) 3D-allele frequency spectra of the empirical data (left) and the best model fit (right) in each pairwise combination between populations. Axes show counts of alleles in each population. Legends indicate number of sites in each cell. See electronic supplementary material, figure S3 for plots including residuals.

#### (ii) Estimating within population genetic diversity

To estimate *θ*_Watterson_, *θ*_π_ and Tajima's D, we mapped reads for each population to an updated *D. montana* PacBio reference genome (N. Poikela & M. Kankare 2020, personal communication) and used NPStat [36] to calculate summary statistics in 1 kb windows (see electronic supplementary material). We averaged windows to obtain scaffold-wide estimates of summary statistics (*n* = 53, representing 95.5% of the genome) and evaluated differences between populations using Kruskal–Wallis rank sum tests followed by pairwise Wilcox rank sum tests corrected for multiple testing using the Benjamini–Hochberg method.

### (c) Measuring postmating prezygotic isolation

If CpSP is present between populations, and results from direct incompatibilities between the male ejaculate and female reproductive tract, then CpSP should be stronger in Colorado females than Vancouver [28,29]. We measured CpSP as male offensive paternity share (*P*_2_) when competing in a foreign or coevolved female reproductive tract against a foreign or coevolved male using the irradiation technique [37]. By mating a virgin female to two males, one of which has been sterilized using irradiation (and controlling for irradiation treatment by performing crosses where the first or second male is irradiated), it is possible to estimate *P*_2_ using equation (1) from Boorman & Parker [37]:

$$P_{R}=\left(1-\frac{x}{p}\right)+\frac{z}{p}*\left(\frac{1-(x/p)}{1-(z/p)}\right),$$

where *x* is the observed proportion of developing eggs after the second mating, *p* is the level of fertility observed in a cross-type where no male is irradiated and *z* is the level of fertility observed in matings with two sterilized males. Males were sterilized using gamma radiation, which renders sperm fertilization competent, yet fertilized eggs will not hatch due to developmental defects in the zygote (see electronic supplementary material). We achieved 100% sterility such that *z* = 0, and the equation can be simplified to $P_{R}=1-x/p$. Therefore, if the irradiated male mates first, then $P_{2}=x/p$. If the irradiated male mates second, then $P_{2}=P_{R}$ [37]. The total number of eggs laid by each female after the second mating was multiplied by the calculated *P*_2_ value and rounded to a whole number to give the estimated number of offspring sired by the second male. The remaining number of eggs expected to hatch were assigned to the first male (see electronic supplementary material).

We assessed differences in hatching (proxy for fertilization) success rates after the first and second mating (for females mated to fertile males only) and differences in *P*_2_ between cross-types using generalized linear models (GLMs) with quasibinomial errors (as model inspection indicated overdispersion). Due to the low fertility in crosses between Colorado females and Vancouver males, we could not estimate *P*_2_ in the CVV cross, which was subsequently excluded from analyses (see electronic supplementary material). We analysed responses in Colorado and Vancouver females separately as populations were never tested together. All statistical analyses were performed in R v. 3.5.1 [38]. Where appropriate, we performed *post hoc* Tukey's honest significant difference (HSD) tests using *glht* [39].

#### (i) Interaction between coevolved and foreign male ejaculates in the female reproductive tract

We tested the prediction that overlapping foreign and coevolved ejaculates within the female reproductive tract would alter PMPZ outcomes by calculating whether observed hatching success rates after a double mating differed from the expected additive effect of two single matings, *H*_total_, using the equation:

$$H_{\mathrm{total}}=({\bar{P}}_{2}\;*\;{\bar{H}}_{2})+(1-{\bar{P}}_{2}\;*\;{\bar{H}}_{1}),$$

where ${\bar{P}}_{2}$ is the mean proportion of offspring sired by the second male in a given cross-type, and ${\bar{H}}_{1}$ and ${\bar{H}}_{2}$ are the mean hatching success after a single mating for a female mated with a male from the first, and second, population denoted in a cross-type, respectively.

### (d) Proxies measuring the intensity of sperm competition within populations

We tested the prediction that PMPZ isolation asymmetries would reflect differences in the intensity of postcopulatory sexual selection acting within populations [16] by measuring two common proxies for the intensity of sperm competition faced by males: male reproductive mass investment and sequential mating capacity [40–42]. Both traits have been shown to be under sexual selection in other *Drosophila* species [43,44]. For detailed methods, see electronic supplementary material.

#### (i) Male relative reproductive investment

We analysed differences between populations in male relative reproductive mass as a measure of total ejaculate investment using analysis of covariance (ANCOVA) [40]. Log transformed dry reproductive tract mass was the response variable, and population identity (Colorado or Vancouver), log transformed dry soma mass (body mass − reproductive tract mass), and their interaction were predictors.

#### (ii) Male mating capacity

We tested for differences between populations in male sequential mating capacity and the total numbers of progeny sired by males across all their mates with Poisson GLMs. To test for differences between populations in male per mating investment we fitted a zero-inflated Poisson GLM using the ‘glmmTMB’ package [45]. Offspring numbers were used as the response variable, with population, mating number, and their interaction as predictors and male identity as a random effect. Two males were lost during the experiment (one Colorado, one Vancouver) and subsequently excluded from analyses.

#### (iii) Female dry mass

We measured dry mass of females as body size is known to correlate with fecundity in *Drosophila* [46]. Females emerging from controlled density vials (*n* = 60 per population) were frozen at −20°C and later thawed and dried overnight at 60°C before being weighed individually on a weighing boat.

## 3. Results

### (a) Demographic modelling

Demographic analyses showed that the best-fitting model included symmetric migration between Oulanka and Vancouver and between Colorado and Vancouver after splitting from the ancestral population, suggesting a demographic history with substantial gene flow between populations (figure 1; table 1; electronic supplementary material, figure S3). The split between Colorado and Vancouver occurred shortly after the North American populations separated from Oulanka (figure 1). Colorado and Oulanka showed larger effective population sizes than Vancouver (table 1). However, the ancestral population size for the North American population before the split was considerably lower, perhaps owing to population contraction following the invasion of North America. For the best-fitting model, parameter estimates show considerably higher migration between the ancestral population and Oulanka (before Colorado and Vancouver split), than between Colorado and Vancouver or Vancouver and Oulanka (figure 1 and table 1). Overall, demographic models with the most support indicate past and/or current gene flow (table 1).

#### (i) Within population genetic diversity

Tajima's D (Kruskal–Wallace test, *χ*^2^ = 57.679, d.f. = 2, *p* < 0.001) and pi (*χ*^2^ = 7.38, d.f. = 2, *p* = 0.025) both showed significant differences between populations, but not Watterson's theta (*χ*^2^ = 4.22, d.f. = 2, *p* = 0.121) (electronic supplementary material, figure S4). All populations showed negative genome-wide Tajima's D, with Colorado and Oulanka exhibiting higher excesses of rare alleles than Vancouver, perhaps owing to recent changes in demography history (e.g. expansion following bottleneck), or selective sweeps. This is supported by higher effective population size estimates in Colorado and Oulanka compared to Vancouver for the best-fitting demographic model (table 1). Pi was significantly greater in Colorado than Oulanka (Wilcoxon rank sum test, *p* = 0.023), suggesting higher genetic diversity in Colorado than Oulanka.

### (b) Postmating prezygotic reproductive isolation

#### (i) Postmating prezygotic isolation of virgin females (non-competitive gametic isolation)

After a single mating, hatching success rates were similar to those reported previously (electronic supplementary material, figure S5) [28,29]. Colorado females had reduced hatching success when mating with Vancouver males relative to mating with Colorado males (quasibinomial GLM: *F*_1,165_ = 341.97, *p* < 0.001, *β* = −3.89 ± 0.28). Vancouver females had reduced hatching success when mating with Colorado males relative to mating with Vancouver males (quasibinomial GLM: *F*_1,136_ = 48.06, *p* < 0.001, *β* = −1.53 ± 0.23).

#### (ii) Postmating prezygotic isolation of non-virgin females

##### Hatching success rates

**Figure 2.**
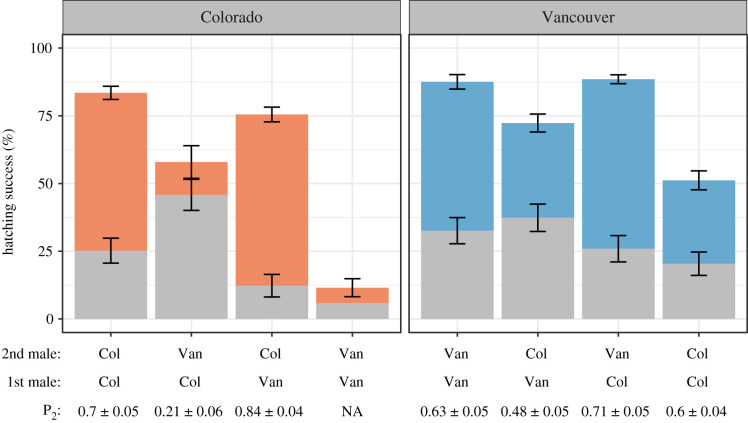
Summary of hatching success and conpopulation sperm precedence (CpSP) for females from Colorado (left panel) and Vancouver (right panel). Full height of each bar represents mean hatching success (% eggs laid that hatched ± standard error) for females that mated with two nonirradiated males. The upper coloured portion of each bar represents the estimated proportion of offspring sired by the second male to mate (*P*_2_ ± standard error), inferred from the irradiated crosses. See electronic supplementary material, figures S6 and S7 for results separately. Note: the cross between Colorado females and two Vancouver males was excluded from *P*_2_ analysis (see methods and electronic supplementary material). Abbreviations: Col, Colorado, Van, Vancouver. (Online version in colour.)

Cross-type had a significant effect on hatching success rates for Colorado females (quasibinomial GLM: *F*_3,111_ = 92.03, *p* < 0.001) and Vancouver females (quasibinomial GLM: *F*_3,137_ = 34.86, *p* < 0.001) after the second mating (figure 2; electronic supplementary material, figure S6). In both populations, females mating with a foreign male followed by a coevolved male had hatching success not significantly different from those females mating with two coevolved males (Tukey's HSD: all *p* > 0.133; mean proportion of eggs that hatched: CCC = 0.84 ± 0.02, *n* = 26; CVC = 0.76 ± 0.03, *n* = 41; VVV = 0.88 ± 0.03, *n* = 29; VCV = 0.89 ± 0.02, *n* = 36). Females mating with two foreign males had lower hatching success than other groups (Tukey's HSD: all *p* < 0.001; CVV = 0.12 ± 0.03, *n* = 30; VCC = 0.51 ± 0.03, *n* = 36). Females mating with a coevolved male followed by a foreign male had lower hatching success than females mating with two coevolved males, but higher hatching success than females mating with two foreign males (Tukey's HSD: all *p* < 0.016; CCV = 0.58 ± 0.06, *n* = 18; VVC = 0.72 ± 0.03, *n* = 40).

##### Conpopulation sperm precedence

Females from both populations showed last-male sperm precedence when mating with two coevolved males (Colorado [CCC], *P*_2_ = 0.70 ± 0.05, *n* = 28; Vancouver [VVV], *P*_2_ = 0.63 ± 0.05, *n* = 35). However, while there was a significant effect of cross-type on *P*_2_ in both Colorado (quasibinomial GLM: *F*_2,83_ = 65.70, *p* < 0.001) and Vancouver (quasibinomial GLM: *F*_3,117_ = 4.93, *p* = 0.003), only Colorado females showed evidence for CpSP (figure 2; electronic supplementary material, figure S7). In Colorado female reproductive tracts Colorado males sired the majority of offspring in both the first (CCV, *P*_2_ = 0.21 ± 0.06, *n* = 25) and second (CVC, *P*_2_ = 0.84 ± 0.04, *n* = 34) mating position. By contrast, Vancouver females mating with a Colorado male followed by a Vancouver male showed *P*_2_ values that were not significantly different from within-population Vancouver matings (VCV, *P*_2_ = 0.71 ± 0.05, *n* = 27). Vancouver females mating with a Colorado male in the second position used sperm equally from the first and second male (VVC, *P*_2_ = 0.48 ± 0.05, *n* = 31; VCC, *P*_2_ = 0.60 ± 0.04, *n* = 28).

#### (iii) Interaction between coevolved and foreign male ejaculates in the female reproductive tract

We found no effect of overlapping foreign and coevolved male ejaculates on fertility. Hatching success calculated for the additive effect of two single matings, given estimated *P*_2_ values, fell within the range of observed hatching success after a double mating (electronic supplementary material, figure S8, and table S2).

### (c) Proxies measuring the intensity of sperm competition within populations

#### (i) Male relative reproductive investment

**Figure 3.**
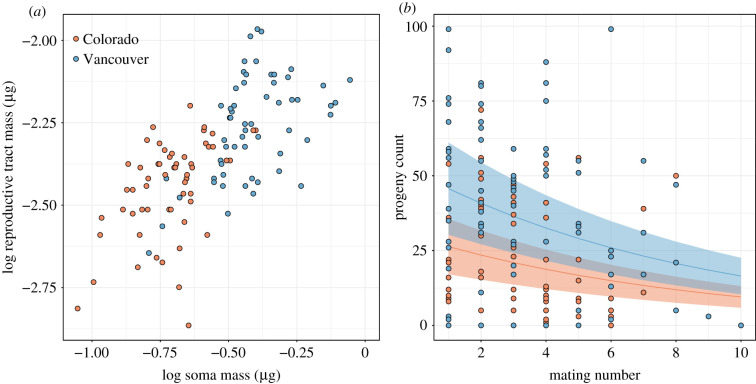
Proxy measures of the intensity of sperm competition experienced by males within populations. Populations did not differ in relative investment in reproductive tract tissue (*a*) or sequential mating capacity and per mating investment (*b*). Colorado, red; Vancouver, blue. Common slopes (±95% confidence intervals) are shown in (*b*). Four outliers were removed in (a) and 2 males were excluded in (*b*).

After dropping the population × soma mass interaction (ANCOVA: *F*_1,112_ = 0.02, *p* = 0.884), the reduced model for male relative reproductive investment showed a significant effect of log soma mass on log reproductive tract mass (*F*_1,113_ = 36.28, *p* < 0.01) but not of population (*F*_1,113_ = 0.62, *p* = 0.433). Log reproductive tract mass increased with log soma mass (figure 3*a*; electronic supplementary material, figure S9). Thus, we found no difference between populations in male relative investment in reproductive tract tissue.

#### (ii) Male mating capacity

Populations did not differ in the number of sequential matings that males initiated (Poisson GLM: *χ*^2^ = 0.01, d.f. = 1, *p* = 0.939; Colorado = 4.42 ± 0.40, *n* = 19; Vancouver = 4.47 ± 0.56, *n* = 19). For male per mating progeny production, there was a significant effect of population (zero-inflated Poisson GLM: *χ*^2^ = 9.81, d.f. = 1, *p* = 0.002) and mating number (*χ*^2^ = 146.2, d.f. = 1, *p* < 0.001). The number of offspring sired declined with mating number (figure 3*b*). The rate of decline was not significantly different between populations (population × mating number interaction, *χ*^2^ = 2.45, d.f. = 1, *p* = 0.118), suggesting that males from Colorado and Vancouver invested similarly per mating. Vancouver males sired more offspring than Colorado males, which resulted in matings within Vancouver producing more offspring than within Colorado overall (quasipoisson GLM: *F*_1,36_ = 6.40, *p* = 0.016; Colorado = 93 ± 16, *n* = 19; Vancouver = 166 ± 25, *n* = 19). Vancouver females weighed more than Colorado females (*t*-test, *t* = −5.81, d.f. = 89.49, *p* < 0.001; Colorado = 0.77 ± 0.01 µg, *n* = 60, Vancouver = 0.92 ± 0.02 µg, *n* = 60). If female body size is indicative of fecundity [46], then this may explain the greater total number of offspring produced in Vancouver crosses.

## 4. Discussion

We used divergent populations of *D. montana* to study the evolution of CSP. We performed demographic modelling which revealed a history of divergence with gene flow between North American and Finnish populations. Further, divergence between Colorado and Vancouver began shortly after the split from the ancestral population. We found CpSP could act as a barrier to gene flow in Colorado females but not Vancouver, showing the same direction of asymmetry to non-competitive PMPZ isolation previously described [28,29]. However, reproductive isolation seems to have evolved in the face of at least some on-going gene flow between these populations. If the strength of selection acting within populations determines the asymmetry of reproductive isolation, then stronger PMPZ isolation in Colorado is expected to be accompanied by heightened postcopulatory sexual selection. However, we found proxies measuring the intensity of sperm competition faced by males did not differ between populations. Finally, while female multiple mating altered reproductive outcomes, we found no evidence of an interaction between foreign and coevolved ejaculates within the female reproductive tract affecting fertilization success.

The demographic modelling suggests divergence between Colorado and Vancouver began shortly after the ancestral North American population split from Oulanka. The phylogeographic history of *D. montana* is uncertain, but it is generally assumed to have arisen in eastern Europe or Asia and invaded North America relatively recently followed by divergence among North American populations during recent ice ages [31]. Our models suggest that the Finnish-North American split occurred approximately 1 750 000 years ago and was followed shortly after by population division within North America (see electronic supplementary material for methods). This is considerably older than previous estimates of the timeframe of the North American-European division in this species, based on mtDNA sequencing [31]. Regardless of actual divergence times, the migration rate estimates suggest gene flow has been on-going during the history of divergence between these populations and, importantly, continued following the splits. Therefore, reproductive barriers do not seem to have appeared only following a cessation of gene flow. Were the populations to become sympatric, such barriers would now contribute to reproductive isolation between them. These inferences of gene flow should be interpreted with caution because, while Pool-seq has been used in similar studies [47,48], the effectiveness in distinguishing gene flow from ancestral allele sharing has not to our knowledge been simulated. Nevertheless, all models including gene flow parameters outperformed those that did not.

Colorado and Oulanka also harboured an excess of rare alleles compared to Vancouver, possibly resulting from population expansions after bottlenecks or selective sweeps. Diminished variance in female preferences and male traits has been hypothesized to result in stronger isolation in bottlenecked populations, whereas genetically diverse populations may be more permissive of a greater diversity of genotypes [49]. We might, therefore, expect Colorado to show lower genetic diversity than Vancouver. However, we found Colorado and Oulanka had larger effective population sizes compared to Vancouver. The most recent genetic analysis identified no fixed SNPs between Colorado and Vancouver [33]. Despite this, the Colorado–Vancouver population pair is the only comparison to show divergence of genes with functional annotations involving reproduction [33] and shows the strongest PMPZ isolation [28,29]. In combination with our results, this shows that specific genes with reproductive function are able to diverge between populations [33] despite the likely presence of gene flow during divergence.

We used the irradiated male technique [37] to determine second male paternity share (*P*_2_), first showing that last-male sperm precedence occurs for both populations (*P*_2_ > 0.63). We subsequently found that CpSP can act as a barrier to gene flow in Colorado because paternity is skewed towards Colorado males when Colorado females mate with Colorado and Vancouver males. By contrast, the Vancouver population did not show evidence of CpSP. When Vancouver females mated with a Colorado male followed by a Vancouver male (VCV) last-male sperm precedence persisted, whereas if the mating order was reversed (VVC) the first and second male sired equal numbers of offspring. Such isolation asymmetries might reflect differences in the strength of postcopulatory sexual selection within populations [16]. However, we found no difference between populations in traits known to evolve in response to the intensity of sperm competition experienced by males [40–42]. This suggests sperm competitiveness alone—at least measured using the proxies we chose—does not predict the strength of PMPZ isolation.

While the proxies for sperm competition we measured have been used before in other species [40–44], other traits may be better proxies in some systems. For example, female remating rate may be a more direct measure of postcopulatory sexual selection [50]. Additionally, sperm length and male accessory gland size both respond to manipulation of the strength of sexual selection [9,44,51]. The pattern of asymmetrical CpSP suggests that Vancouver males can maintain sperm offensiveness against a Colorado male ejaculate in Vancouver female reproductive tracts but cannot maintain a sperm defensive role. In *D. melanogaster*, longer and slower sperm are better able to retain representation in the fertilization set [52]. In *Drosophila*, female sperm storage organs can exert selection on sperm length as a form of cryptic female choice [51] and sperm and female sperm storage organ length show correlated evolution between populations [51,53] and between species [54]. Sperm–female reproductive tract length coevolution within populations, possibly driven by cryptic female choice, could lead to mismatches between populations resulting in PMPZ isolation [5,14]. There are two alternatives: (i) postcopulatory sexual selection is not important in driving PMPZ isolation in *D. montana* or (ii) CpSP could result from ‘mutation-order’ divergence, whereby traits (in this case ejaculate × female reproductive tract interactions) diverge along different, perhaps arbitrary, evolutionary trajectories due to the stochastic nature in which mutations arise and fix [19].

Finally, we tested whether foreign and coevolved ejaculates interacting in the female reproductive tract altered PMPZ outcomes. Mating with two foreign males produced a similar pattern of PMPZ isolation to that of a single mating, where mating with a foreign male is particularly costly for Colorado females [28,29]. The cost of between-population mating was reduced when females mated with both a foreign and coevolved male. However, we found no effect of overlapping foreign and coevolved male ejaculates on hatching success beyond the expected additive effect of two single matings. This pattern suggests that the ejaculates of the two males act independently and supports an interpretation that PMPZ isolation is at least in part a consequence of cryptic female choice and that this plays a more important role than ejaculate × ejaculate interactions in determining PMPZ outcomes in *D. montana*.

To conclude, we found divergence between European and North American *D. montana* populations was followed shortly after by divergence within North America. CpSP was stronger in Colorado than Vancouver despite their more recent divergence, and probably evolved in the face of on-going gene flow. CpSP shows asymmetry in the same direction as non-competitive PMPZ isolation, suggesting a similar mechanism underlies these phenomena. We show that CpSP can evolve between populations, but apparently not in a way predicted by either time since divergence or the strength of sperm competition acting within populations. Male ejaculate × female reproductive tract interactions are complex traits that evolve rapidly and divergently [7,55]. The Colorado–Vancouver population pair shows low genome-wide divergence, yet divergence in genes involved in reproduction [33]. While we examine only two populations in one species, the evolutionary processes that can cause such rapid and unpredictable divergence between populations, such as sexual coevolution within populations, are common to many taxa. Perhaps such postmating incompatibilities are a potential example of mutation-order divergence and hence would occur sporadically and unpredictably, suggesting PMPZ isolation could be more prevalent than is currently documented.

## Supplementary Material

Supplementary material

## Data accessibility

Data and code are available from the Dryad Digital Repository: https://dx.doi.org/10.5061/dryad.gqnk98sjd [56] and github: https://github.com/MartinGarlovsky/conpopulation-sperm-precedence.
