# Supplementary material for: Within-population sperm competition intensity does not predict asymmetry in conpopulation sperm precedence
Source: Philos Trans R Soc Lond B Biol Sci. 2020 Oct 19;375(1813):20200071. doi: 10.1098/rstb.2020.0071 (PMC7661444; doi:10.1098/rstb.2020.0071)
Supplement: Supplementary material [file rstb20200071supp1.pdf]

Within-population sperm competition intensity does not predict asymmetry in conpopulation sperm precedence: supplementary material

Martin D. Garlovsky[1*], Leeban H. Yusuf[2], Michael G. Ritchie[2] & Rhonda R. Snook[3]

June 2020

[1]Department of Animal and Plant Sciences, University of Sheffield, Sheffield S10 2TN, UK
[*]Current address: Department of Biology, Syracuse University, Syracuse NY 13244, USA
[2]Centre for Biological Diversity, University of St. Andrews, Fife KY16 9TH, UK
[3]Department of Zoology, Stockholm University, Stockholm 106-91, Sweden
Corresponding author: martingarlovsky@gmail.com,

ORCiD: MDG: 0000-0002-3426-4341 LHY: 0000-0001-6383-1176 MGR: 0000-0001-7913-8675 RRS: 0000-0003-1852-1448

Contents

Population genetics **2**
DNA sequencing . . . 2
Demographic modelling . . . 2
Estimating within population genetic diversity . . . 6

Measures of postmating prezygotic reproductive isolation **6**
Postmating prezygotic isolation of virgin females: non-competitive gametic isolation (NCGI) . . . 7
Postmating prezygotic isolation of non-virgin females . . . 8
Con-population sperm precedence (CpSP) . . . 8
Interaction between coevolved and foreign male ejaculates in the female reproductive tract . . . 10

Proxies for the intensity of sperm competition faced by males **12**
Male relative reproductive investment . . . 12
Male mating capacity . . . 15

References **16**

This markdown document provides supplementary material for Garlovsky, M. D., Leeban H. Yusuf, Michael G. Ritchie and Rhonda R. Snook "Within-population sperm competition intensity does not predict asymmetry in conpopulation sperm precedence". Complete analyses and data are available via Dryad and github.

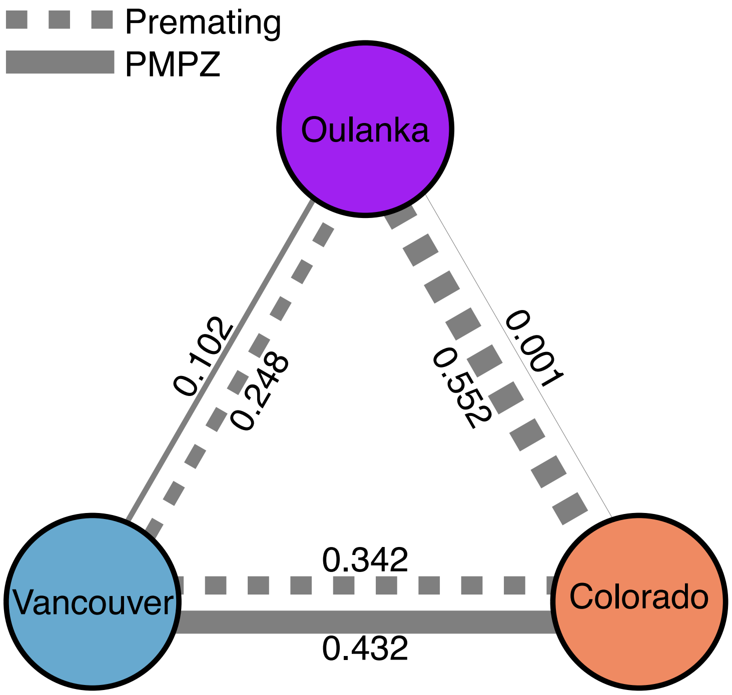

Figure S1: Summary of prezygotic isolation between populations of *Drosophila montana* from Crested Butte, Colorado, USA, Oulanka, Finland, and Vancouver, Canada. Edge weights reflect absolute contributions of premating sexual isolation (dashed) and non-competitive PMPZ isolation (solid) to total reproductive isolation between each pair of populations (from Table 3 in Jennings et al. (2014)).

Population genetics

DNA sequencing

Genomic DNA was extracted from pooled samples of 50 females per population and sequenced using an Illumina HiSeq 2000 to produce paired-end reads (90 + 90 bp, insert size = 500 bp) at the Beijing Genomics Institute (Parker et al. 2018). Reads were trimmed, filtered and down-sampled to ensure equal representation of each population (Parker et al. 2018). Sequencing was performed in 2013, 5 years after collection for Oulanka and Vancouver, and 6 years for Colorado (around 50 generations).

Demographic modelling

All single nucleotide polymorphisms (SNPs) were filtered using a minimum coverage of 30 and we randomly subsampled SNPs to end with a uniform coverage of 30. To ensure SNPs included in the analysis were unlikely to be linked, we then randomly down-sampled based on how far SNPs were apart by selecting 1 in every 100 SNPs.

We indexed the *D. montana* Illumina reference genome and mapped Pool-seq reads using BWA-MEM v.0.75 (Li 2013). We sorted reads, removed duplicates, and converted mapped reads using SAMtools v.1.6 (Li et al. 2009) and Popoolation2 (Kofler et al. 2011). All single nucleotide polymorphisms (SNPs) were filtered using a minimum coverage of 30 and we randomly subsampled SNPs to end with a uniform coverage of 30 before converting read counts into a format suitable for $\delta a \delta i$ (Diffusion Approximation for Demographic Inference. To satisfy the assumption of independence between SNPs, we down-sampled from 860,228 to 8,000 SNPs by selecting 1 in every 100 SNPs post-filtering to ensure SNPs in close proximity were not used in the demographic analysis.

We tested seven demographic models for the history of divergence between *D. montana* populations from Crested Butte, Colorado, USA, Oulanka, Finland, and Vancouver, Canada (Fig. S2). (1) a no-migration model where migration was not included; (2) a full-symmetrical migration model where migration was symmetrical and present between all populations; (3) an adjacent-migration model where migration was possible between Oulanka and Vancouver or between Colorado and Vancouver, but not between Colorado and Oulanka. Additionally, we included models incorporating periods of isolation falling under two categories: ancient migration models and secondary contact models. Ancient migration models included (4) a model assuming migration ceased at the time of the Colorado-Vancouver split, and (5) a model assuming migration persisted after the Finnish-North American split and the Colorado-Vancouver split but ceased shortly after. For secondary contact models, we included (6) a model with isolation between populations with a recent secondary contact event between Colorado and Vancouver after divergence, and (7) a model assuming migration during the Colorado-Vancouver divergence, with a period of secondary contact beginning after the appearance of both Colorado and Vancouver populations. Migration is assumed to be symmetric for all models.

We fitted all seven models to the 3D-AFS and performed 3 rounds of model optimizations using the Nelder-Mead method (Nelder and Mead 1965). In the first round, minimum (0.01) and maximum (10) parameters were 3-fold perturbed with 10 replicates and a maximum of 5 iterations. The best fitting parameters from the first round were used as starting parameters for the second round, in which parameters were 2-fold perturbed with 10 replicates and a maximum of 5 iterations. Finally, the best fit replicate from the second round was used to produce starting parameters for the third round, in which we ran 15 replicates with parameters perturbed 1-fold with a maximum of 5 iterations.

We estimated divergence times by first solving the equation for θ, the population mutation rate, to derive N_{ref}, the reference effective population size:

$$\theta = 4N_{ref}\mu L, \tag{1}$$

where μ is the mutation rate for *D. melanogaster* (2.8x10^{-9}) (Keightley et al. 2014) and L, the effective length was calculated by multiplying the total number of SNPs aligned to the reference genome and converted to sync format (31,800,188), by the number of SNPs that entered the analysis divided by the number of SNPs remaining before down-sampling (8,000/860,228). We then converted parameter estimates to real biological units of time, τi, using the equation:

$$\tau i = 2\tau 1 N_{ref} G, \tag{2}$$

where G, the generation time, was set as one per year based on observations from Aspi et al. (1993) and Throckmorton (1982).

Using equations (1) and (2) we estimated divergence from the ancestral population to have occurred 1,743,164 years ago (T1 + T2), and between Colorado and Vancouver shortly after (T2 = 1,710,355 years ago). We advise caution when interpreting these estimates however. Despite studies showing accurate allele-frequency estimation using Pool-seq (Futschik & Schlötterer 2010; Gautier et al. 2013), it is difficult to appreciate how the use of read count data derived from a DNA pool might affect demographic inference and divergence time estimates. Here, for example, we observe some evidence of an excess of rare variants in our data, though it is unclear whether this represents demographic and/or selective processes, or inaccurate allele frequency estimation caused by inherent statistical challenges associated with Pool-seq. To account for this, we stringently filtered and down-sampled our dataset to retain only reliable sites. Nonetheless, an excess of rare variants may have biased the demographic inference performed here. Additionally, the generation time (G), effect seqeunce length (L) and mutation rate (μ) used, whilst these are reasonable assumptions, may have also contributed to over- or underestimation of divergence times.

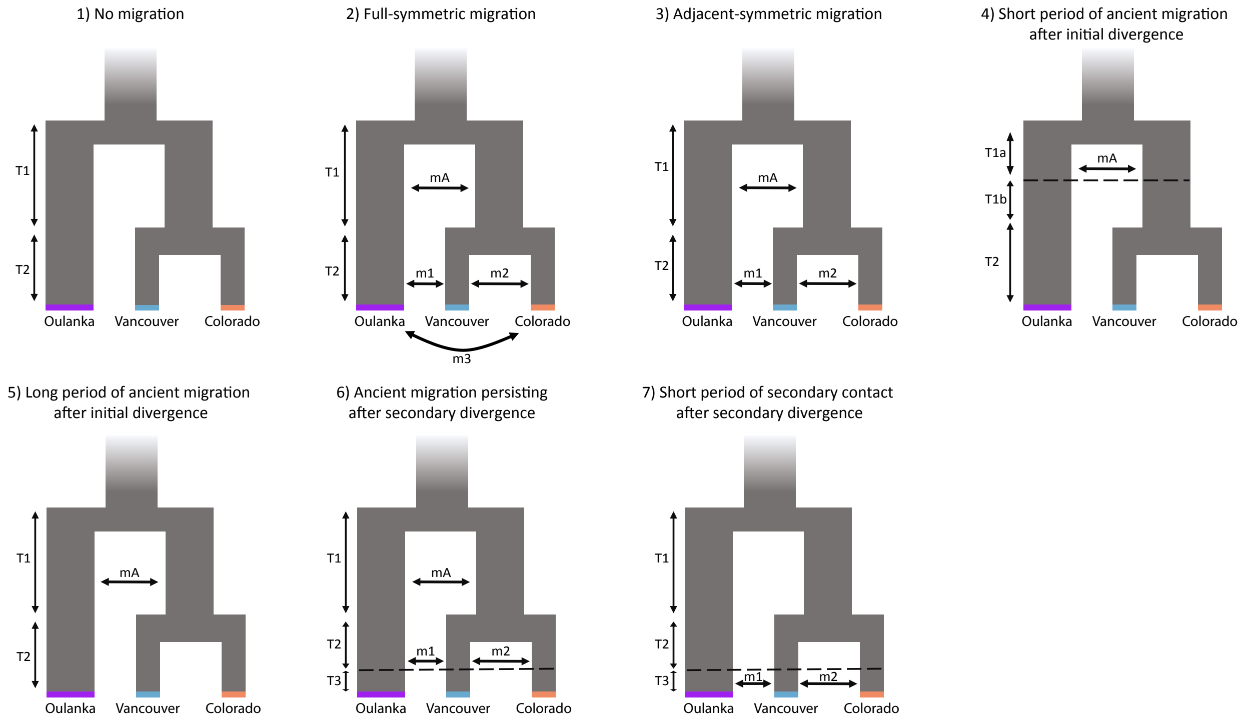

Figure S2: Graphical representation of demographic models tested. mA, ancient migration between Oulanka and precursor North American population; m1, ancient migration between Vancouver and Oulanka; m2, ancient migration between Vancouver and Colorado; m3, ancient migration between Oulanka and Colorado; Initial and secondary divergence are represented by T1(a) and T2, where T1(b) and T3 correspond to the start or end of periods of isolation. Population splits are denoted by T1 (between Oulanka and North America) and T2 (between Vancouver and Colorado). T1(a) and T1(b) denote the start or end of periods of migration following the split between Oulanka and North American populations. T3 denotes start or end of periods of migration following the split between Vancouver and Colorado.

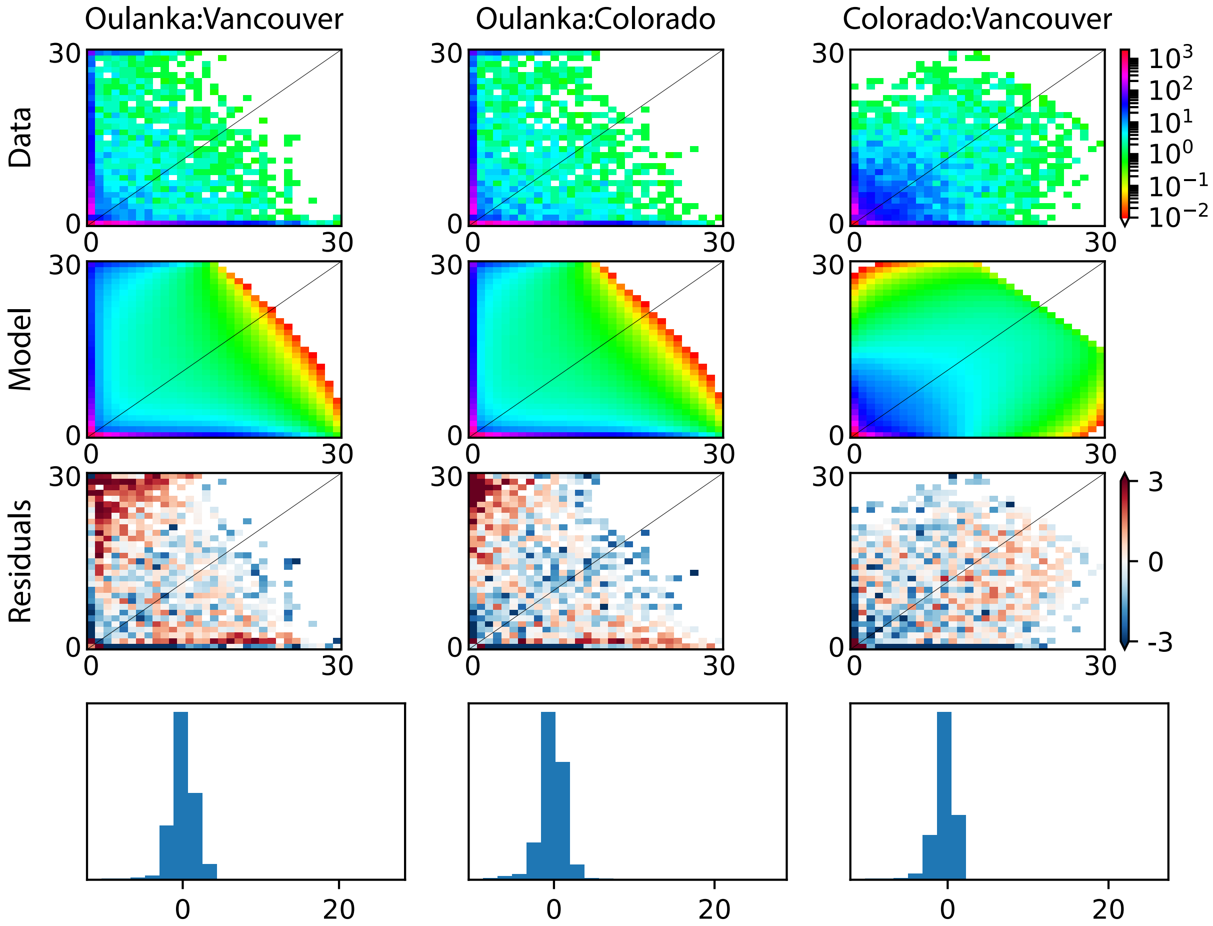

Figure S3: Three-dimensional allele-frequency spectra (3D-AFS) for the best fitting model; (3) Adjacent-migration model. Top: data; middle top: model fits; middle bottom: residuals; bottom: histogram of residuals.

Estimating within population genetic diversity

We mapped reads for each population to an updated *D. montana* PacBio reference genome supplied by colleagues (Poikela et al. in prep.) as above using BWA-MEM and conversion to pileup using SAMtools. To estimate $\theta_{Watterson}$, θ_{π} and Tajima's D, we used NPStat v.1 (Ferretti et al. 2013) and calculated summary statistics in 1kb windows, with minimum coverage (10), maximum coverage (100), minimum base quality (30) and minimum allele count (2) filters. We averaged windows for each of 53 scaffolds (representing 95.5% of the genome) to obtain scaffold-wide estimates of summary statistics and evaluated differences between populations using Kruskal-Wallis rank sum tests followed by pairwise Wilcox rank sum tests corrected for multiple testing using the Benjamini-Hochberg method.

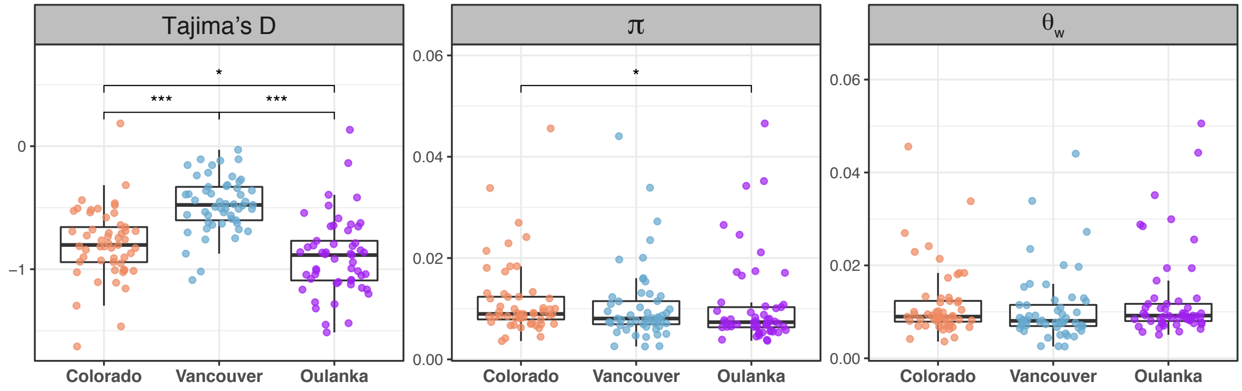

Figure S4: Genome-wide estimates of Tajima's D, pi (π), and Watterson's theta (θ_W) calculated in 1kb windows. Points show the mean for each scaffold, n = 53 in each case. Pairwise differences between populations were evaluated using Wilcox rank sum tests corrected for multiple testing using the Benjamini-Hochberg method: * $p < 0.05$, ** $p < 0.01$, *** $p < 0.001$.

Measures of postmating prezygotic reproductive isolation

We measured PMPZ isolation between Colorado and Vancouver using hatching success rates (a proxy for fertilisation success) (Jennings et al. 2014; Garlovsky and Snook 2018) in single and double matings, and assessed CpSP by estimating male offensive paternity share (P2) using the irradiated male technique (Boorman and Parker 1976). We performed experiments in 3 blocks for each focal female population separately, in which virgin females (n = 10 per cross-type per treatment per block) were presented with a virgin male from Colorado or Vancouver and allowed a 4-hour mating opportunity. After mating, males were discarded and females were transferred to a chamber in an oviposition manifold, which allows easy counting of numbers of eggs oviposited and assessment of hatching success (Jennings et al. 2014; Garlovsky and Snook 2018). Manifolds were returned to the incubator for oviposition. Two days later, females were removed from their chambers, and placed in a vial with a second virgin male from Colorado or Vancouver and allowed a 4-hour remating opportunity. Remating females were returned to their chambers, placed back into the incubator and allowed to oviposit for a further two days on a new oviposition plate. We ensured no females mated more than once during either the first or second observation period. To measure hatching success the numbers of eggs laid on each oviposition plate were counted immediately after removing the female (for remating or discarding). The oviposition plate was then returned to the incubator for a further two days after which the numbers of unhatched eggs was counted again.

We assessed differences between cross-types in hatching success rates after the first and second mating (for females mated to fertile males only) using generalized linear models (GLMs) with binomial errors and a logit link, where the numbers of hatched ("successes") and unhatched ("failures") eggs was the response variable and the only predictor was the cross-type. We assessed differences in P2 using GLMs with binomial errors and a logit link where the binary response was the numbers of offspring sired by the second male ("successes") and numbers of offspring sired by the first male ("failures") (see supplementary material). Models included cross-type, irradiation order and their interaction as predictors. Females that did not remate, died prior to

remating, or laid less than 10 eggs were excluded. Due to the low fertility in crosses between Colorado females and Vancouver males, we could not estimate P2 in the CVV cross, which was subsequently excluded from analyses (see supplementary material). We analysed responses in Colorado and Vancouver females separately as populations were never tested together. All statistical analyses were performed in R v.3.5.1 (R Core Team 2018). We used quasi-errors for GLMs where preliminary model inspection indicated overdispersion. Where appropriate we performed post-hoc Tukey's honest significant difference (HSD) tests using the glht function from the 'multcomp' package (Hothorn et al. 2008).

Postmating prezygotic isolation of virgin females: non-competitive gametic isolation (NCGI)

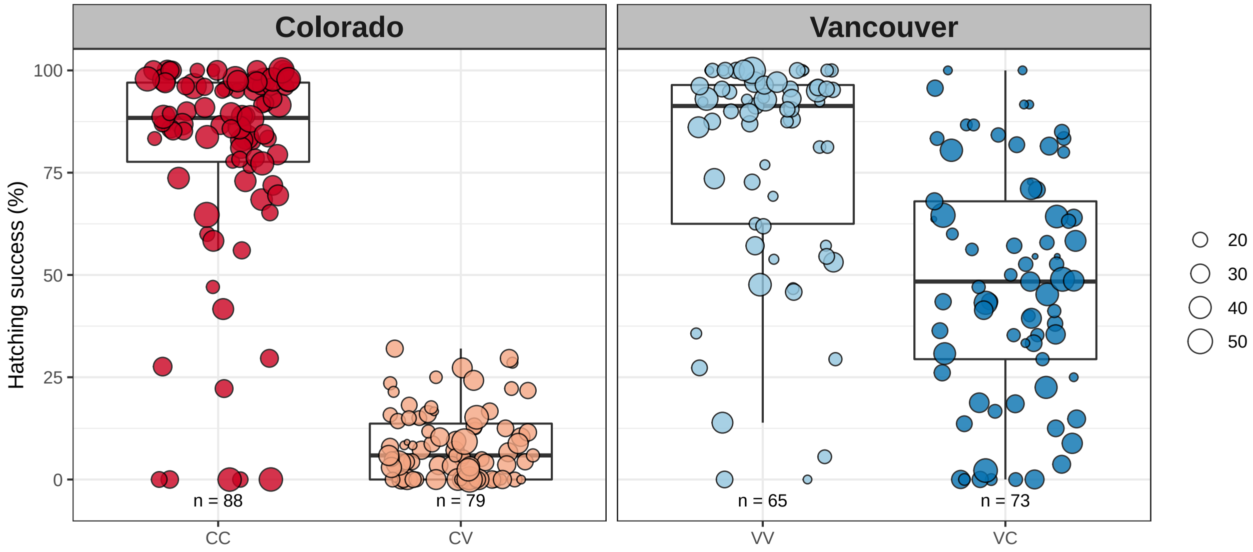

Figure S5: Hatching success (% eggs laid that hatched) after the first mating for females that mated with nonirradiated males. Points are observations and size show the number of eggs laid (i.e. weights). Cross-type denotes female population followed by first and second male. C, Colorado; V, Vancouver.

Postmating prezygotic isolation of non-virgin females

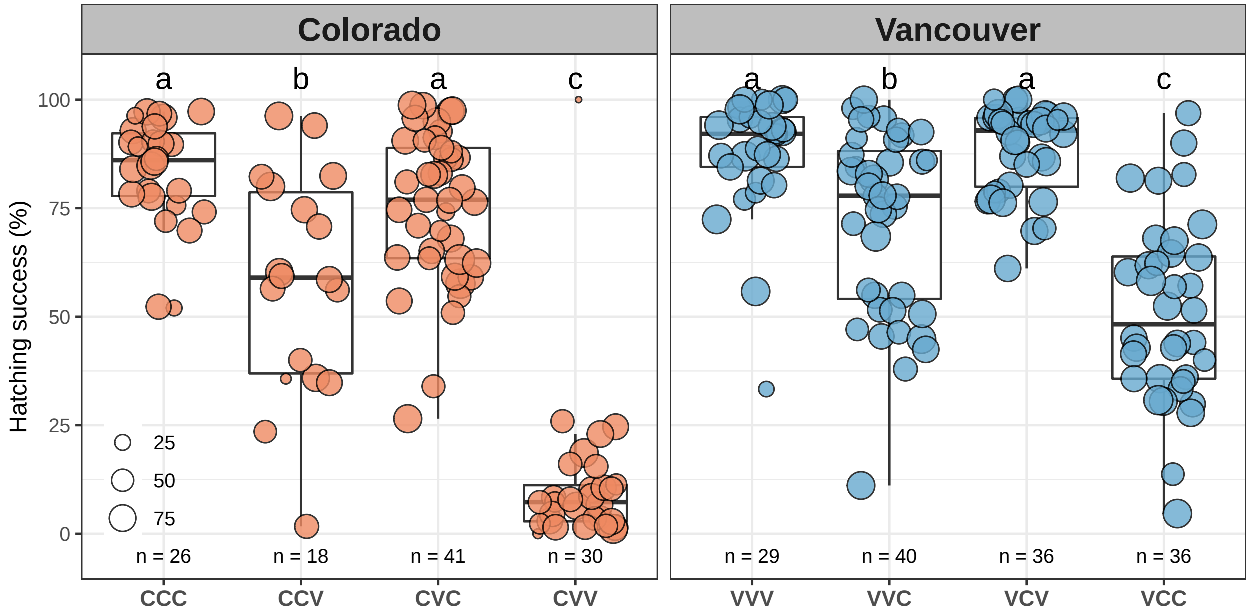

Figure S6: Hatching success (% eggs laid that hatched) after the second mating for females that mated with nonirradiated males. Points are observations and size show the number of eggs laid (i.e. weights). Cross-type denotes female population followed by first and second male. C, Colorado; V, Vancouver.

Con-population sperm precedence (CpSP)

We assessed male offensive paternity share (P_2) using the irradiated male technique (Boorman and Parker 1976). Virgin males were irradiated with a 100Gy dose of gamma radiation (dose rate 189.2 rads min^{-1}, ^{137}Cs source), rendering males 100% sterile, less than 24 hours before mating and housed individually overnight in food vials. To assess the level of fertility after a double mating, 'control' treatments consisted of mating females with two nonirradiated males in all possible crossing combinations with males from Colorado and/or Vancouver. We also included additional data for these control crosses collected in a pilot study (n = 30 per cross-type). To assess the efficacy of the irradiation technique females were mated with two irradiated males, both from Colorado or Vancouver. Experimental treatments consisted of all possible crossing combinations between females and males from Colorado and Vancouver, with either the first or second male irradiated. The proportion of eggs fertilised by the irradiated male after the second mating, P_R, was calculated using equation (1) from Boorman and Parker (1976):

$$P_R = \left(1 - \frac{x}{p}\right) + \left(\frac{x}{p} * \frac{1 - (x/p)}{1 - (z/p)}\right), \tag{3}$$

where x is the observed proportion of developing eggs after a double mating from the second oviposition plate, p is the level of fertility observed in a double mating for a given 'control' cross-type, and z is the level of fertility observed in the double irradiated cross-type. For our calculation of P_R we chose the maximum value of p observed (rather than the mean) to capture the full potential fertility of between-population crosses. As $z = 0$ in this case, the equation can be simplified to $P_R = 1 - x/p$. Therefore, if the irradiated male mates first, then the proportion of eggs fertilised by the second male, $P_2 = x/p$. If the irradiated male mates second then $P_2 = P_R$ (Boorman and Parker 1976). The total number of eggs laid by each female after the second mating was multiplied by the calculated P_2 value and rounded to a whole number to give the estimated number of offspring sired by the second male. The remaining number of eggs expected to hatch were assigned to the first male.

As few eggs laid hatch when Colorado females mate with Vancouver males (CV hatching success = 0.084 ± 0.01 [mean ± standard error], n = 79, Fig. S5; CVV hatching success = 0.115 ± 0.033, n = 30, Fig. S6), we could not estimate differences in P_2 for the CVV cross-type which was subsequently excluded from P_2 analyses (Fig. S7).

Table 1: Mean P_2 with standard errors. Value calculated for CVV cross is included for completeness. C, Colorado; V, Vancouver. N = sample size.

Cross-type	N	P_2	Std. Err.
CCC	28	0.70	0.05
CCV	25	0.21	0.06
CVC	33	0.84	0.04
CVV	25	0.49	0.09
VVV	35	0.63	0.05
VVC	31	0.48	0.05
VCV	27	0.71	0.05
VCC	28	0.60	0.04

Irradiation order effects on P_2

In Colorado there was a significant effect of irradiation treatment ($F_{1,82} = 20.41$, p < 0.001), and the cross-type x irradiation interaction on P_2 ($F_{2,80} = 14.12$, p < 0.001). Irradiated males mating in the second position had a greater P_2 than irradiated males mating in the first position in the CCV and CVC cross-types, but not the CCC cross-type (Fig. S7). In Vancouver the irradiation main effect was not significant ($F_{1,116} = 1.24$, p $= 0.267$) but there was a significant cross-type x irradiation interaction ($F_{3,113} = 7.37$, p < 0.001). In the VVV cross-type irradiated males in the second position had lower P_2, whereas in the other Vancouver female cross-types irradiated males in the second position had greater P_2 (Fig. S7).

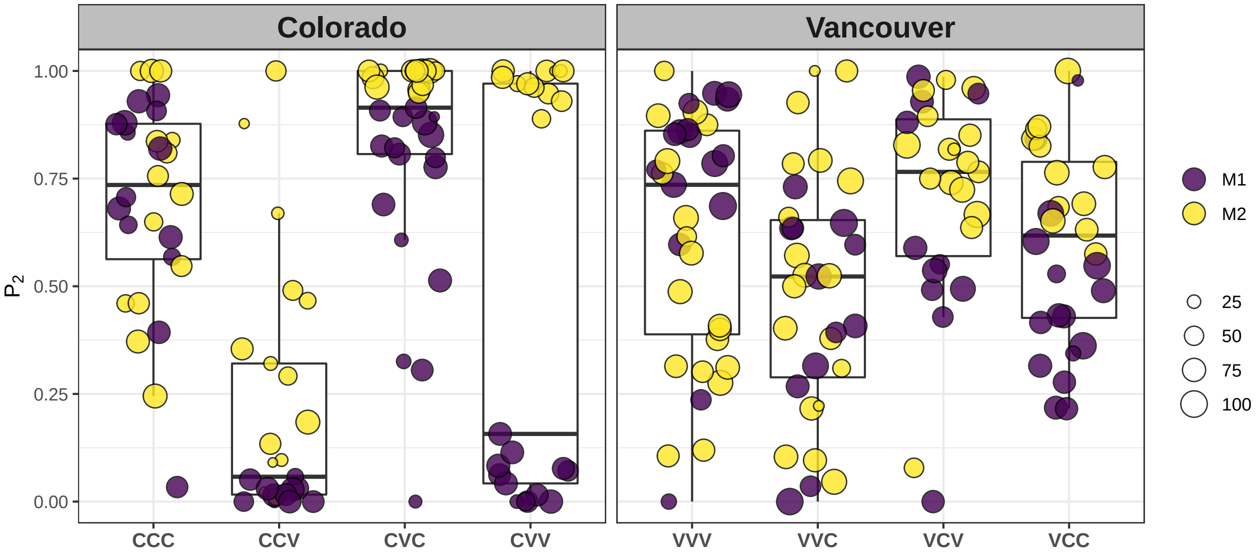

Figure S7: Proportion of offspring sired by the second male to mate (P_2), where either the first (M1, purple) or second (M2, yellow) male to mate was irradiated. Cross-type denotes female population followed by first and second male mate. C, Colorado; V, Vancouver. Points are observations and size show the number of eggs laid (i.e. weights).

Interaction between coevolved and foreign male ejaculates in the female reproductive tract

We tested whether observed hatching success rates after a double mating in the nonirradiated 'control' crosses differed from the expected additive effect of two single matings, H_{total}, using the following equation:

$$H_{total} = (P_2 * H_2) + (1 - P_2 * H_1)\,, \tag{4}$$

where P_2 is the mean proportion of offspring sired by the second male in a given cross-type and H_1 and H_2 are the mean hatching success rates after a single mating for a female mated with a male from the first, and second, population denoted in a cross-type, respectively (Table 2).

Table 2: Values used to calculate H_{total}, the expected additive effect of two single matings using equation (4). Cross-type denotes female population followed by first and second male mate. C, Colorado; V, Vancouver.

Cross-type	P_2	H_{obs}	H_1	H_2	RHS	LHS	H_{total}	Δ
CCC	0.70	0.83	0.80	0.80	0.24	0.56	0.80	0.03
CCV	0.21	0.58	0.80	0.08	0.63	0.02	0.65	-0.07
CVC	0.84	0.75	0.08	0.80	0.01	0.67	0.68	0.07
CVV	0.49	0.12	0.08	0.08	0.04	0.04	0.08	0.03
VVV	0.63	0.88	0.78	0.78	0.29	0.49	0.78	0.09
VVC	0.48	0.72	0.78	0.48	0.41	0.23	0.64	0.09
VCV	0.71	0.88	0.48	0.78	0.14	0.55	0.69	0.19
VCC	0.60	0.51	0.48	0.48	0.19	0.29	0.48	0.03

P_2, mean second male paternity share; H_{obs}, mean observed hatching success for females that mated with two nonirradiated males; H_1, mean hatching success from a single mating expected for females mating with first male; H_2, mean hatching success from a single mating expected for females mating with second male; RHS, $1 - P_2 * H_1$; LHS, $P_2 * H_2$; H_{total}, LHS + RHS; $\Delta = H_{obs} - H_{total}$

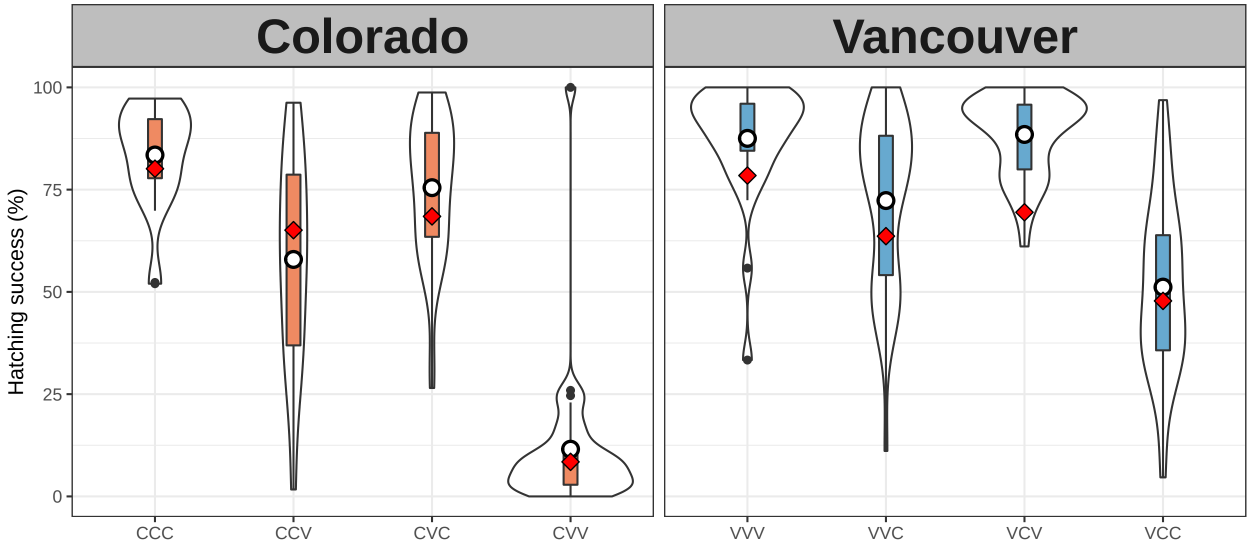

Figure S8: Effects of overlapping foreign and coevolved ejaculates on fertilisation success. White points show H_{obs}, mean hatching succcess (% eggs laid that hatched) for females that mated with two nonirradiated males (data same as Fig. S6), red diamonds show H_{total}, the expected additive hatching success of two single matings for a female mated with a male from the first and second population denoted in a cross-type, calculated given the estimated paternity share of each male (Table 2). Cross-type denotes female population followed by first and second male mate. C, Colorado; V, Vancouver.

Proxies for the intensity of sperm competition faced by males

Male relative reproductive investment

We analysed differences between populations in male relative reproductive mass as a measure of total ejaculate investment (Tomkins and Simmons 2002). Approximately 100 flies of mixed sex were collected from population cages and allowed to mate and oviposit for two days in plastic bottles covered with a molasses-agar oviposition plate and a drop of dried yeast paste. Controlled density vials (CDVs) containing food were seeded with 50 first instar larvae from oviposition plates. Adults were collected from CDVs on the day of eclosion and kept in single sex vials until sexually mature and then frozen at -20ºC. Population identity was replaced with a unique identifier prior to measurement. Males (n = 60 per population) were thawed and the entire reproductive tract (testes, accessory glands, ejaculatory duct and bulb) dissected on a pre-weighed piece of foil in a drop of dH20 which was then transferred to a second piece of pre-weighed foil. Carcasses and reproductive tracts were dried overnight at 60ºC before weighing (METTLER TOLEDO® UMX2 ultra-microbalance).

We analysed differences between populations in male relative reproductive investment using analysis of covariance (ANCOVA) (Tomkins and Simmons 2002). Log transformed dry reproductive tract mass was regressed against log transformed dry soma mass (body mass – reproductive tract mass) and the soma mass x population interaction. Colorado males weighed significantly less than Vancouver males on average (Welch Two Sample t-test, t = -9.84, df = 96.45, $p < 0.001$; Colorado = 0.58 ± 0.01 mg [mean ± standard error], n = 60, Vancouver = 0.77 ± 0.02 mg, n = 60; Fig. S9a). Colorado males reproductive tracts weighed significantly less than Vancouver males on average (Welch Two Sample t-test, t = -6.47, df = 114.14, $p < 0.001$; Colorado = 0.09 ± 0.002 mg, n = 60, Vancouver = 0.11 ± 0.002 mg, n = 60; Fig. S9c). The results of the ANCOVA revealed the population x log soma mass interaction was not significant (ANCOVA: $F_{1,116} = 0.995$, p = 0.321) and so was dropped from the model. The reduced model showed a significant effect of population (ANCOVA: $F_{1,117} = 7.74$, p = 0.006) and log soma mass (ANCOVA: $F_{1,117} = 8.63$, p = 0.004) on reproductive mass. Log reproductive tract mass increased with log soma mass and Vancouver males had a higher intercept (Fig. S9b). However, visual inspection of model diagnostic plots revealed four consistent outliers (Fig. A1). One Vancouver male had an unusually small body mass which resulted in high leverage (Fig. S9a). Three males (2 Colorado, 1 Vancouver) had relatively small reproductive tract masses (Fig. S9c).

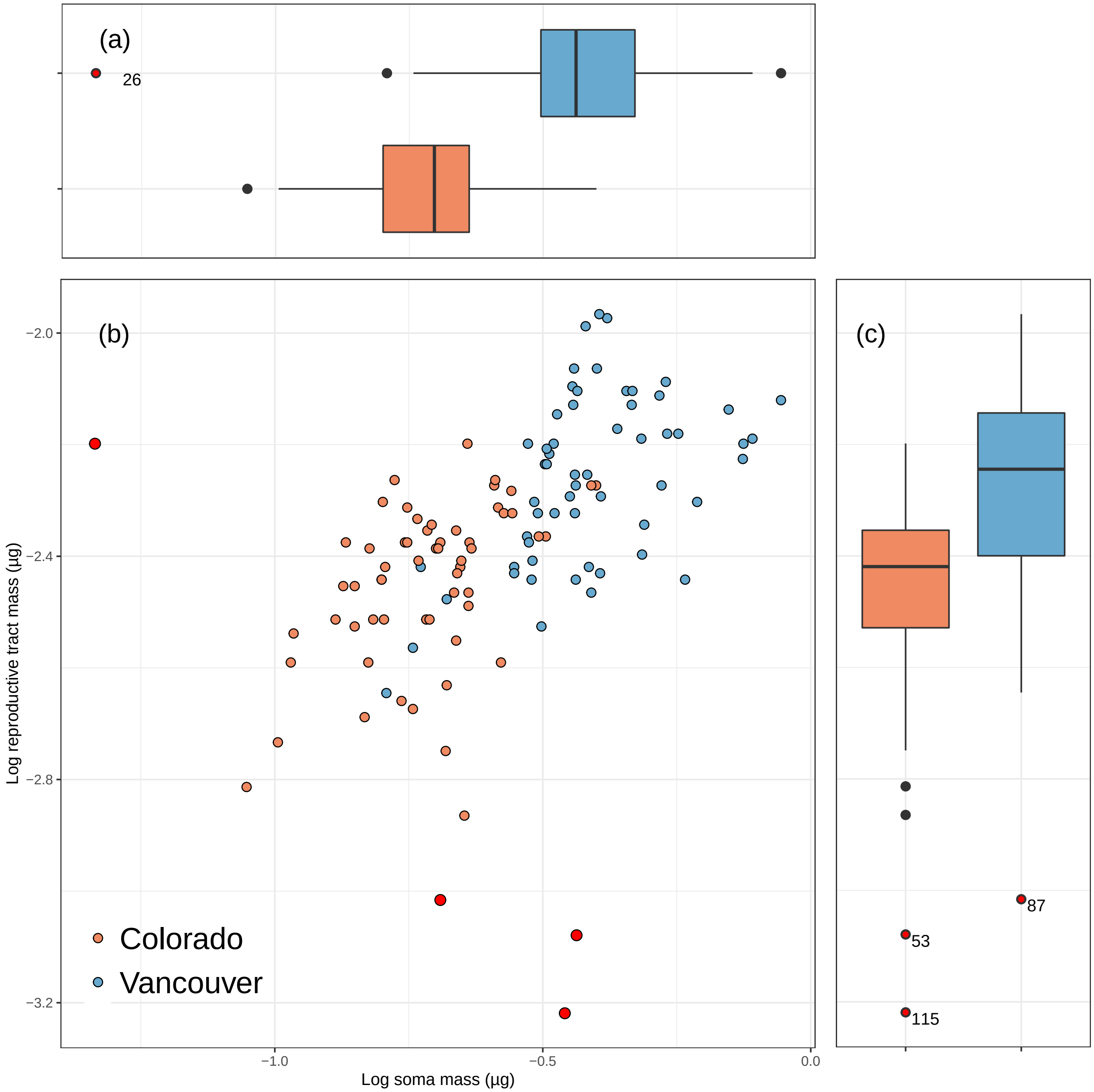

Figure S9: Log reproductive tract mass increased with log soma mass (ANCOVA: common slope, $\beta = 0.520 \pm 0.086$, $t = 6.023$, $p < 0.001$). Red, Colorado; Blue, Vancouver. Outliers removed for analysis (2 Colorado, 2 Vancouver) are numbered and shown in red.

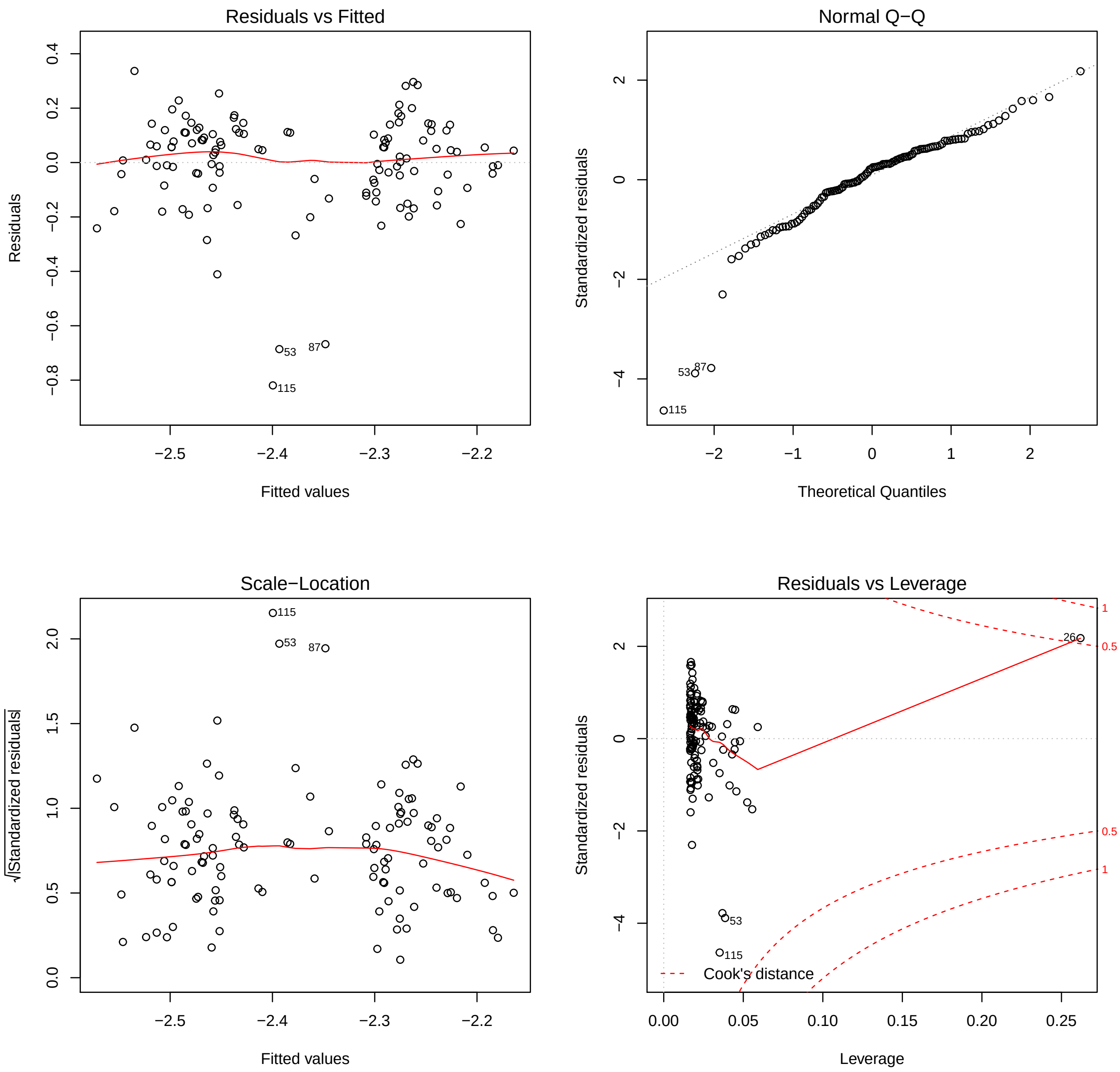

Figure A1: Model diagnostic plots for ANCOVA after dropping the population x soma mass interaction including all data. Outliers are numbered as in Fig. S9.

After removing the four outliers, Colorado males still weighed significantly less than Vancouver males on average (Welch Two Sample t-test, t = -11.36, df = 98.8, p $<$ 0.001) and Colorado reproductive tracts weighed significantly less than Vancouver males on average (Welch Two Sample t-test, t = -6.91, df = 106.27, p $<$ 0.001). Results of the updated ANCOVA model again revealed that the population x log soma mass interaction was not significant (ANCOVA: $F_{1,112} = 0.02$, p = 0.884), and was subsequently dropped from the model. Visual inspection of model diagnostic plots showed no significant outliers (Fig. A2).

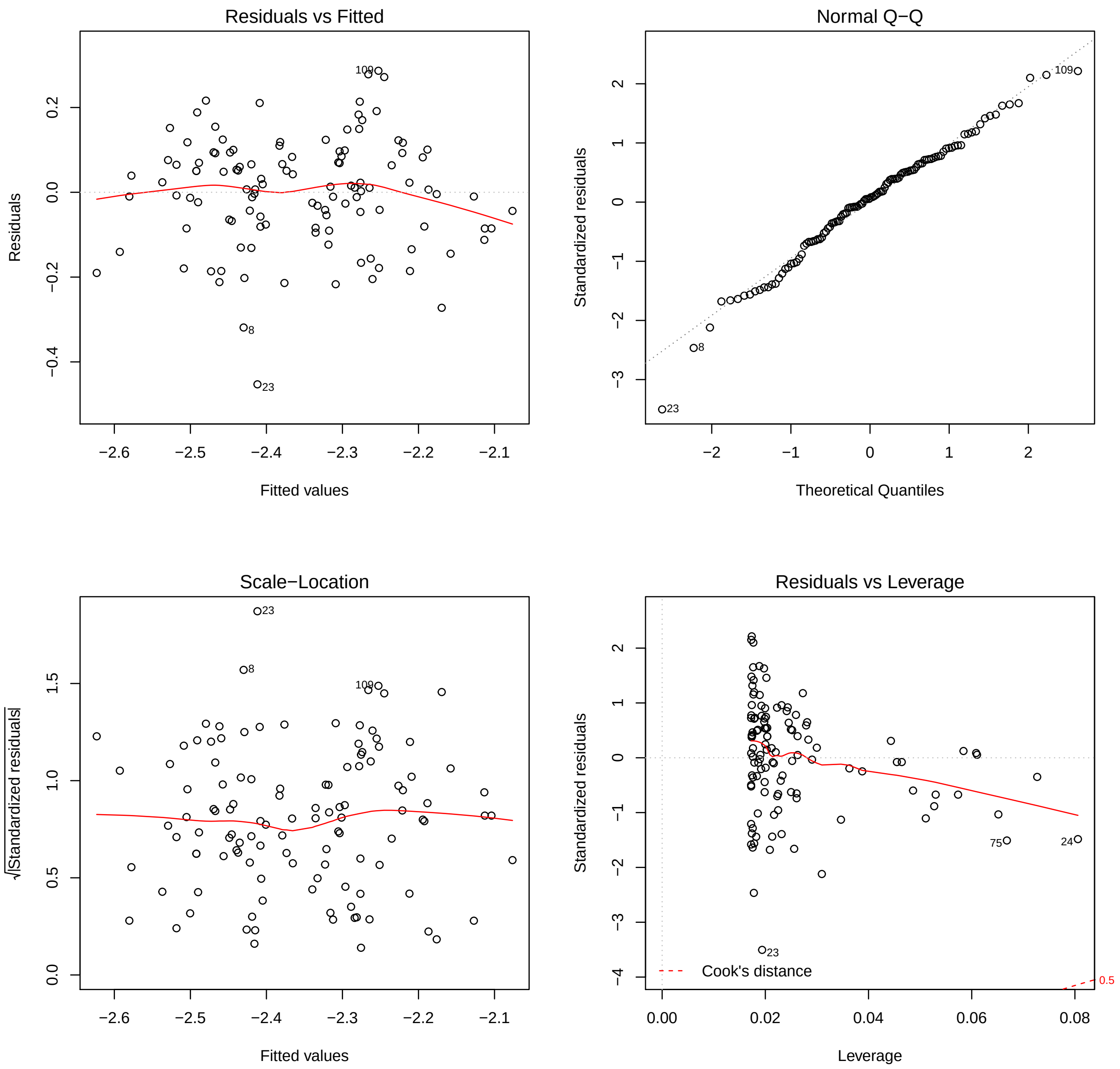

Figure A2: Diagnostic plots for ANCOVA after dropping the population x soma mass interaction model after excluding outliers.

Male mating capacity

Virgin males (n = 20 per population) were mouth aspirated without anaesthesia into a vial containing two virgin females from their own population. Once copulation began unmated females were removed from the vial. The male was transferred to a new vial housing another two virgin females after mating ad libitum and we recorded the total number of matings that males performed within a 4-hour period. Mated females were returned to the incubator in their oviposition vial for 4 days, before being transferred to a second food vial for a further 4 days. The total number of adult flies emerging was counted for each female (combined across both oviposition vials).

References

- Aspi J, Lumme J, Hoikkala A, Heikkinen E. 1993 Reproductive ecology of the boreal riparian guild of *Drosophila.* Ecography 16, 65–72.
- Boorman E, Parker GA. Sperm (ejaculate) competition in *Drosophila melanogaster*, and the reproductive value of females to males in relation to female age and mating status. Ecol Entomol. 1976 Aug 1;1(3):145–55.
- Ferretti, L., S. E. Ramos-Onsins, and M. Pérez-Enciso. 2013. Population genomics from pool sequencing. Mol. Ecol., doi: 10.1111/mec.12522.
- Futschik A, Schlötterer C. 2010 The next generation of molecular markers from massively parallel sequencing of pooled DNA samples. Genetics 186, 207–218. (doi:10.1534/genetics.110.114397)
- Garlovsky, M. D., and R. R. Snook. 2018. Persistent postmating, prezygotic reproductive isolation between populations. Ecol. Evol. 8:9062–9073.
- Gautier M et al. 2013 Estimation of population allele frequencies from next-generation sequencing data: Pool-versus individual-based genotyping. Mol. Ecol. 22, 3766–3779. (doi:10.1111/mec.12360)
- Hothorn, T., F. Bretz, and P. Westfall. 2008. Simultaneous inference in general parametric models. Biom. J. 50:346–363.
- Jennings JH, Snook RR, Hoikkala A. Reproductive isolation among allopatric *Drosophila montana* populations. Evolution. 2014 Nov 1;68(11):3095–108.
- Keightley PD, Ness RW, Halligan DL, Haddrill PR. 2014 Estimation of the spontaneous mutation rate per nucleotide site in a *Drosophila melanogaster* full-sib family. Genetics 196, 313–320. (doi: 10.1534/genetics.113.158758)
- Li, H. 2013. Aligning sequence reads, clone sequences and assembly contigs with BWA-MEM. ArXiv13033997 Q-Bio.
- Li, H., B. Handsaker, A. Wysoker, T. Fennell, J. Ruan, N. Homer, G. Marth, G. Abecasis, and R. Durbin. 2009. The Sequence Alignment/Map format and SAMtools. Bioinformatics 25:2078–2079.
- Nelder JA, Mead R. A Simplex Method for Function Minimization. Comput J. 1965 Jan 1;7(4):308–13.
- Parker, D. J., R. A. W. Wiberg, U. Trivedi, V. I. Tyukmaeva, K. Gharbi, R. K. Butlin, A. Hoikkala, M. Kankare, M. G. Ritchie, and J. Gonzalez. 2018. Inter and intraspecific genomic divergence in *Drosophila montana* shows evidence for cold adaptation. Genome Biol. Evol. 10:2086–2101.
- R Core Team. 2018. R: A language and Environment for Statistical Computing. R Foundation for Statistical Computing, Vienna, Austria.
- Tomkins JL, Simmons LW. Measuring relative investment: a case study of testes investment in species with alternative male reproductive tactics. Anim Behav. 2002 May 1;63(5):1009–16
- Throckmorton LH. 1982 The virilis species group. In The genetics and biology of *Drosophila*, London: Academic press.
